# Genomic Differences between Listeria monocytogenes EGDe Isolates Reveal Crucial Roles for SigB and Wall Rhamnosylation in Biofilm Formation

**DOI:** 10.1128/JB.00692-19

**Published:** 2020-03-11

**Authors:** Chih-Yu Hsu, Lynne Cairns, Laura Hobley, James Abbott, Conor O’Byrne, Nicola R. Stanley-Wall

**Affiliations:** a Division of Molecular Microbiology, School of Life Sciences, University of Dundee, Dundee, United Kingdom; b Data Analysis Group, Division of Computational Biology, School of Life Sciences, University of Dundee, Dundee, United Kingdom; c Bacterial Stress Response Group, Microbiology, School of Natural Sciences, National University of Ireland Galway, Galway, Ireland; Ohio State University

**Keywords:** *Listeria monocytogenes*, biofilm formation, sigma B, rhamnose, cell wall teichoic acid, biofilms, genome analysis

## Abstract

Listeria monocytogenes is a Gram-positive firmicute that causes foodborne infections, in part due to its ability to use multiple strategies, including biofilm formation, to survive adverse growth conditions. As a potential way to screen for genes required for biofilm formation, we harnessed the ability of bacteria to accumulate mutations in the genome over time, diverging the properties of seemingly identical strains. By sequencing the genomes of four laboratory reference strains of the commonly used L. monocytogenes EGDe, we showed that each isolate contains single nucleotide polymorphisms (SNPs) compared with the reference genome. We discovered that two SNPs, contained in two independent genes within one of the isolates, impacted biofilm formation. Using bacterial genetics and phenotypic assays, we confirmed that *rsbU* and *rmlA* influence biofilm formation. RsbU is the upstream regulator of the alternative sigma factor SigB, and mutation of either *rsbU* or *sigB* increased biofilm formation. In contrast, deletion of *rmlA*, which encodes the first enzyme for TDP-l-rhamnose biosynthesis, resulted in a reduction in the amount of biofilm formed. Further analysis of biofilm formation in a strain that still produces TDP-l-rhamnose but which cannot decorate the wall teichoic acid with rhamnose (*rmlT* mutant) showed that it is the decorated wall teichoic acid that is required for adhesion of the cells to surfaces. Together, these data uncover novel routes by which biofilm formation by L. monocytogenes can be impacted.

**IMPORTANCE** Biofilms are an important mode of growth in many settings. Here, we looked at small differences in the genomes of the bacterium Listeria monocytogenes isolate EGDe and used them to find out how biofilms form. This important fundamental information may help new treatments to be developed and also highlights the fact that isolates of the same identity often diverge.

## Abstract (precis)

Biofilms are an important mode of growth in many settings. Here, we looked at small differences in the genomes of the bacterium Listeria monocytogenes isolate EGDe and used them to find out how biofilms form. This important fundamental information may help new treatments to be developed and also highlights the fact that isolates of the same identity often diverge.

## INTRODUCTION

Biofilms are complex communities of microbial cells that are encased within a self-produced extracellular matrix. The biofilm matrix provides protection from environmental insults, increasing the tolerance of cells to antimicrobial agents and biocides (1). Listeria monocytogenes is a Gram-positive bacterium that causes the foodborne infection listeriosis. In susceptible individuals (e.g., people who are immunocompromised), the mortality rate of *Listeria* infections has been estimated to be up to 30%. Biofilms of L. monocytogenes can form on machinery in food-processing plants, contributing to food contamination (2) and potentially leading to the closure of manufacturing facilities for deep-clean processes (3). Thus, routes to inhibit or disrupt biofilm formation by L. monocytogenes could present one means of reducing *Listeria* infections. It is currently known that biofilm formation by L. monocytogenes is dependent on an active flagellum (4). Moreover, two major transcription factors, SigB and PrfA, and the virulence factor ActA have been shown to contribute to biofilm formation (5–7). However, there are still many unanswered questions regarding the molecular processes underpinning L. monocytogenes biofilm formation.

Reference strains of bacteria are widely used in laboratories as research models for the study of bacterial behavior and physiology (8). However, mutations can be inadvertently introduced into the genome during routine culture, modifying the strains derived from the designated laboratory reference strain (9). Diverging mutations within laboratory reference strains can contribute to differences in observed phenotypic behavior between different research groups. For example, Bacillus subtilis laboratory reference strain 168 was identified as a nonrugose biofilm-forming strain (10); however, it has been shown that some variants can form biofilms (11). By sequencing a collection of 12 sublines of strain 168, it was revealed that the *epsC* gene, which is essential for biofilms, carried point mutations in the nonrugose biofilm isolates. L. monocytogenes EGDe, serovar 1/2a, is widely used for molecular and cellular studies as the model organism (12), and we chose to use this isolate in our studies. We predicted that if we were able to identify genomic variations between L. monocytogenes isolates used by different laboratories, this could potentially shed light on the underlying genetics of biofilm formation. Using a comparative sequencing approach, we identified and connected genomic variations in L. monocytogenes EGDe isolates with differences in biofilm formation. More specifically, our bioinformatic analysis and experimental approaches revealed two genes, *rsbU* and *rmlA*, involved in biofilm formation. This work contributes to our understanding of biofilm formation by an important human pathogen.

(Data included in this article have been published in Chih-Yu Hsu’s doctoral thesis [53].)

## RESULTS

### Assessing growth and flagellum-based motility.

**FIG 1:**
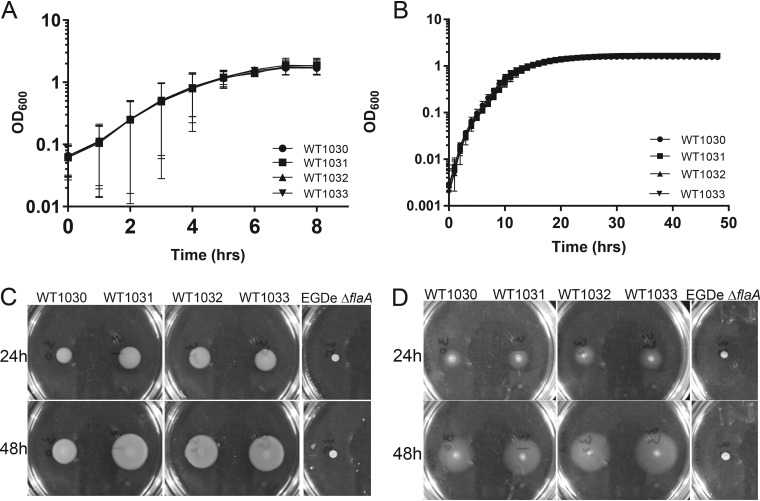
Growth and motility of the four L. monocytogenes EGDe isolates. (A) Growth in BHI medium under shaking conditions at 37°C. (B) Growth in MWB under static conditions at 30°C. The values presented in panels A and B are the means from 2 independent experiments, and the error bars represent the standard deviations. (C and D) Motility of the four isolates assessed after 24 and 48 h at 30°C using BHI (C) or MWB (D) soft agar. The EGDe Δ*flaA* strain was used as a negative control. Representative images are presented.

Four different L. monocytogenes EGDe isolates were obtained for this study and are here referred to as WT_1030_, WT_1031_, WT_1032_, and WT_1033_ (Table 1). The designation of the bacteria used in the study as EGDe was initially based on information obtained from the source supplying them and was later confirmed by whole-genome sequencing. We first compared the growth rates and yields of all strains and assayed motility. Growth was monitored under shaking culture conditions using brain heart infusion (BHI) medium and under static culture conditions using modified Welshimer’s broth (MWB). We did not identify any statistically significant differences in the growth rates or final yields of the four isolates for either condition (Fig. 1A and B). Next, we assessed flagellum-based motility by quantifying the ability of the cells to spread on semisolid agar plates, using EGDe Δ*flaA* as a negative control (13). The four EGDe isolates and EGDe Δ*flaA* were spotted onto BHI- and MWB-based semisolid agar plates that were incubated at 30°C, the permissive temperature for motility by L. monocytogenes (13, 14). As expected, the EGDe Δ*flaA* strain did not spread from the inoculation point (Fig. 1C and D). In contrast, the four EGDe stocks spread from the seeding point over time, although WT_1030_ showed reduced motility on BHI agar by comparison to the other three isolates (Fig. 1C and D; see Fig. S1 in the supplemental material). These data indicate that any differences in biofilm formation observed are not due to impaired growth or mutation of the flagellar genes.

**TABLE 1:** The Listeria monocytogenes EGDe isolates used in this study

Strain	Reference^*a*^	Origin
WT_1030_	ANG882	Carmen Buchrieser via Angelika Gründling
WT_1031_	ANG873	Martin Loessner via Angelika Gründling
WT_1032_	EGDe	University College Cork
WT_1033_	BAA-679	Carmen Buchrieser via ATCC

a The strain name used in the originating lab.

### Differences in chitinase activity.

**FIG 2:**
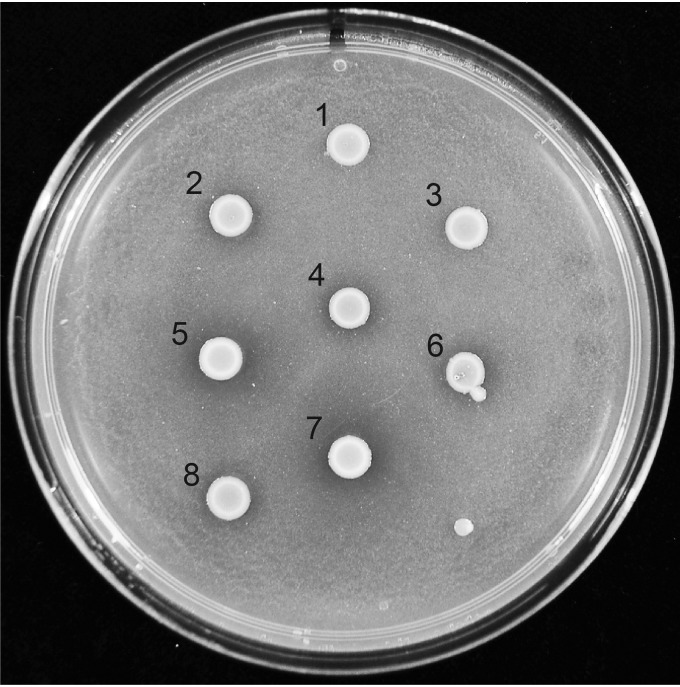
Chitinase activity of the four L. monocytogenes EGDe isolates. Chitinolytic activity assessed using LB agar containing 2% (wt/vol) chitin. Incubation was at 30°C for 120 h. The genotypes of the strains tested are as follows: 1 and 3, 1031 Δ*sigB*; 2 and 4, 1031 Δ*rsbU*; 5, WT_1031_; 6, WT_1030_; 7, WT_1032_; 8, WT_1033_. The 1031 Δ*sigB* and 1031 Δ*rsbU* strains were used as controls.

Certain regions of the L. monocytogenes genome are prone to incorporating mutations during growth (15), including *rsbS*, *rsbU*, and *rsbV* (16). The products of these genes comprise part of the complex regulatory system that activates the alternative sigma factor sigma B (SigB) (17). In turn, SigB controls a large regulon in L. monocytogenes that includes the genes *chiA* and *chiB*, which encode extracellular chitinases (18). Thus, to test if SigB regulation was disrupted due to mutations in the *rsb* genes, we examined chitinase activity (16). After spotting the four EGDe isolates onto chitin-rich agar, we noted that two of the isolates displayed clear evidence of chitinolytic activity: WT_1031_ and WT_1032_. In contrast, colonies formed by WT_1030_ and WT_1033_ had less distinct clearance zones, suggesting altered expression of members of the SigB regulon (Fig. 2). These gross phenotypic differences are indicative of genomic variations existing between the four EGDe isolates.

### Whole-genome sequencing.

We next sequenced the genomes of the EGDe strains using Illumina next-generation technologies. The reads were mapped to the published wild-type EGDe reference genome (NC_003210), and single nucleotide polymorphisms (SNPs) were identified in each of the four strains by using variant detection (Table 2). Some of the SNPs initially identified (not shown in Table 2) in the WT_1032_ genome were close to the prophage A118 integration site; further bioinformatic analysis revealed that these were caused by excision of the prophage from the chromosome, restoring a functional copy of *comK* (19, 20). Isolates WT_1030_ and WT_1033_ both contained a nonsense SNP in *rsbU*; this is consistent with the chitinase analyses which showed that these isolates generated a less distinct clearance zone on chitin-containing growth medium. WT_1031_ contained the fewest SNPs, all of which were identified in the other EGDe isolates, and so was designated the parental “wild-type” strain. These findings support the conclusion that variations in the genome have emerged between the EGDe isolates obtained from different sources.

**TABLE 2:** Analysis of single nucleotide polymorphisms using whole-genome sequencing data

Relative position in genome^*a*^	Gene	Ref^*b*^	SNP					Alteration of amino acid^*c*^	Type of mutation^*d*^
			WT 1030	WT 1031	WT 1032	WT 1033	EGDe Δ*flaA*		
188308	*lmo0184*	G	T	—^*e*^	—	T	—	148 E to stop codon	Nonsense
189757	*lmo0185*	C	A	—	—	—	—	—	Synonymous
264578	*lmo0247*	G	T	T	T	T	T	—	Synonymous
280225	*rpoC*	C	G	—	—	—	—	1166 I to M	Missense
435968	Intergenic	C	A	A	A	A	A	Intergenic	Intergenic
929469	*rsbU*	C	CTT	—	—	CTT	—	245 L to F, frameshift	Nonsense
1116367	*lmo1081 (rmlA)*	G	T	—	—	—	—	241 E to stop codon	Nonsense
1442124	Intergenic	C	A	A	A	A	A	Intergenic	Intergenic
1890030	*lmo1814*	C	A	—	—	A	—	82 G to W	Missense
2003900	*aroF*	C	A	—	—	—	—	138 V to F	Missense
2207164	*lmo2125*	T	G	—	—	—	—	400 Q to P	Missense
2734614	*lmo2660*	C	A	—	—	—	—	211 G to V	Missense
2836724	*lmo2757*	G	—	—	A	—	—	354 R to C	Missense
2849710	*lmo2769*	G	—	—	—	T	—	247 Y to stop codon	Nonsense
2943565	Intergenic	G	T	T	T	T	T	Intergenic	Intergenic

a The relative locations of the SNPs present in the strains are compared with NC_003210.

b Ref, nucleotide present at the corresponding relative position in NC_003210.

c The codons of the coding sequences with SNPs were analyzed by ExPASy translation tool followed by BLAST with the original amino acid sequences.

d SNPs were categorized as intergenic, synonymous, missense, or nonsense.

e —, no difference from the reference genome.

### Biofilm formation by the L. monocytogenes EGDe stocks.

**FIG 3:**
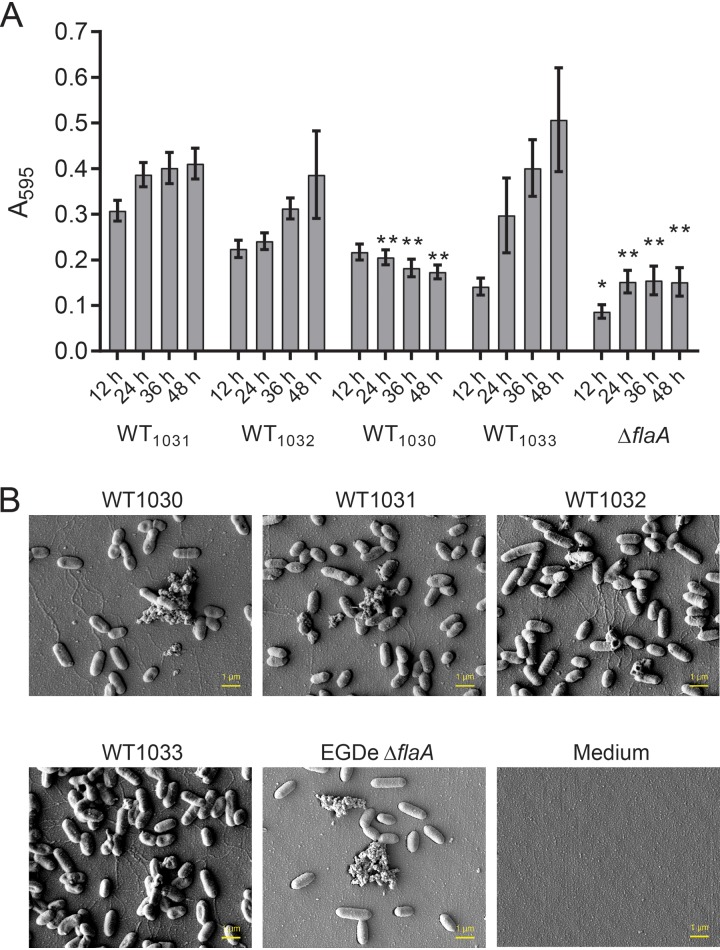
Biofilm formation of the four L. monocytogenes EGDe isolates. (A) The biomasses of the four EGDe isolates adherent to the substratum were quantified over time when incubated at 30°C. The EGDe Δ*flaA* strain was used as a negative control. The values presented are the means from 29 independent experiments for the EGDe isolates and 4 experiments for the Δ*flaA* strain. The error bars are the standard errors of the means. The data were analyzed by one-way ANOVA comparing with WT1031. *, *P* ≤ 0.05; **, *P* ≤ 0.01. (B) The biomass adherent to the substratum was imaged using scanning electron microscopy after 48 h of incubation. The representative images shown were taken at the midpoint of the peg.

Having identified that the genomes of the four EGDe isolates were nonidentical, we assessed biofilm formation. The four EGDe stocks were inoculated in a 96-well microtiter plate platform where polystyrene pegs protruded from the lid into the well (this is also known as a Calgary biofilm device [21]). The EGDe Δ*flaA* strain, which was previously shown to be impeded in biofilm formation (4), was included as a negative control. The cultures were incubated statically at 30°C, and the biomass of each biofilm was measured every 12 h for a 48-h period. As expected, EGDe Δ*flaA* exhibited lower *A*_595_ readings then those of the four EGDe stocks (Fig. 3A), indicative of biofilm formation being reduced. Using the data from WT_1031_ as a baseline, the profiles of biofilm biomasses measured for the other three EGDe isolates were found to differ (Fig. 3A). Overall, the biomass of WT_1030_ was lower at all time points (Fig. 3A), whereas the biomass of WT_1033_ started at a lower point than WT_1031_ but ended with higher measurements at later time points (Fig. 3A). The statistical analysis revealed the measurements for WT_1032_ to be comparable to those of the reference WT_1031_ (Fig. 3A). The findings indicate that excision of prophage A118 does not impact biofilm formation as assessed here.

We next imaged the adherent cells by using scanning electron microscopy (Fig. 3B). This analysis was conducted at 30°C after biofilms were grown for 48 h. Five regions of interest (ROI) were chosen for each sample that covered the top (liquid surface) to near to the bottom of the peg (Fig. S2A). We first compared the overall cell morphology of the EGDe isolates and concluded that there were no discernible differences (Fig. 3B). We next counted the individual cells per field of view (FOV), and in doing so, we noticed that dense aggregates of cells encased in extracellular material were only encountered infrequently for all of the strains. The biomass produced by WT_1031_ contained on average ∼810 ± 320 (mean ± standard deviation [SD]) cells per FOV (Fig. S2B). Moreover, consistent with the measurements derived from crystal violet staining, the number of cells per FOV calculated for WT_1032_ did not significantly vary from those measured for WT_1031_. In contrast, fewer cells were counted per FOV for WT_1030_, while considerably more cells were detected in the WT_1033_ samples (1,255 ± 539). It is worth noting that in some cases, the cell density per FOV seemed to change with the location on the peg; the region of the peg that was closer to the bottom of the well had a higher number of cells than an equivalent region nearer the liquid-air interface (Fig. S2C). This gradient of cell attachment was most apparent for the biofilms formed by WT_1033_ (Fig. S2C). In summary, the biomasses measured using crystal violet and by counting the adherent cells per FOV correlate well.

### Linking genotype and biofilm formation.

Our data suggest that WT_1030_ is impeded in biofilm formation by comparison with WT_1031_, a phenotype that is a consequence of fewer cells attaching to the substratum. As detailed in Table 2, the WT_1030_ genome contains 6 missense SNPs and three nonsense SNPs. To identify which of these mutations was responsible for reducing cell attachment, we constructed single gene deletions in the coding regions that contained nonsense SNPs, *lmo0184*, *rmlA* (*lmo1081*), and *rsbU*, using WT_1031_ as the parent. We reasoned that the nonsense SNPs were more likely to have a significant impact on protein function than the missense SNPs and, additionally, links to biofilm formation can be made for both *rsbU* and *rmlA* (22).

**FIG 4:**
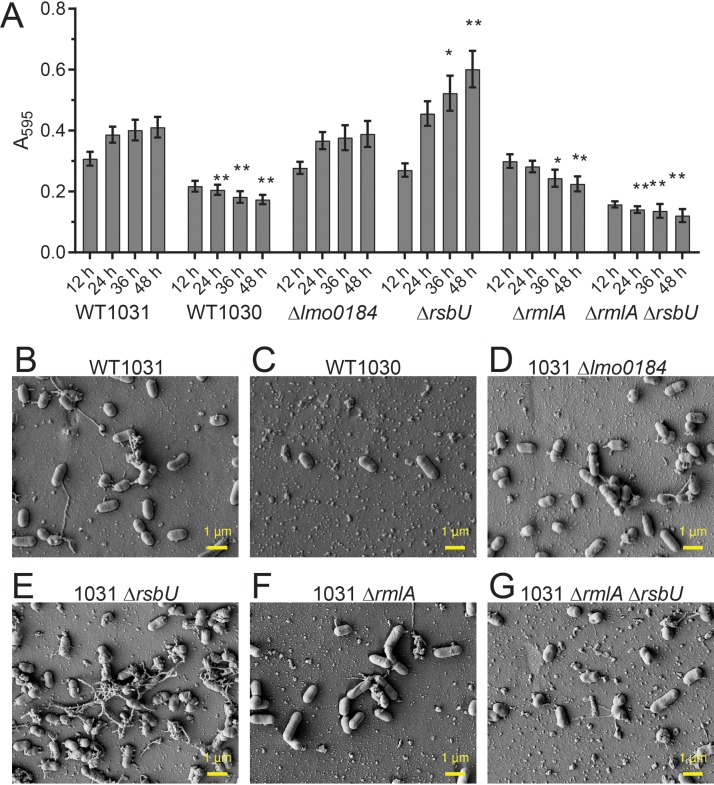
RmlA and RsbU influence biofilm formation by L. monocytogenes EGDe. (A) The biomasses of WT_1031_, WT_1030_, WT_1031_ Δ*lmo0184* (LSW1024), WT_1031_ Δ*rsbU* (LSW1028), WT_1031_ Δ*rmlA* (LSW1040), and WT_1031_ Δ*rmlA* Δ*rsbU* (LSW1051) strains that were adherent to the substratum were quantified. The samples were incubated at 30°C for the time points indicated. The values presented for WT_1031_ and WT_1030_ are reproduced from Fig. 3. The means from ≥4 experiments are presented for the remaining strains. The error bars are the standard errors of the means. The data were analyzed by one-way ANOVA, comparing with WT1031. *, *P* ≤ 0.05; **, *P* ≤ 0.01. The biomasses adherent to the substratum were imaged using scanning electron microscopy for WT_1031_ (B), WT_1030_ (C), WT_1031_ Δ*lmo0184* (D), WT_1031_ Δ*rsbU* (E), WT_1031_ Δ*rmlA* (F), and WT_1031_ Δ*rmlA* Δ*rsbU* (G). The representative images shown were taken at the midpoint of the peg after 48 h of incubation.

We checked if planktonic growth of the deletion strains was different from that of the parental strain WT_1031_ (Fig. S3). No significant differences were detected. Next, we measured the biomass adhered to the pegs of the Calgary biofilm device for the deletion strains by using crystal violet staining. We discovered that deletion of *lmo0184* did not impact biofilm formation compared with that of WT_1031_ (Fig. 4A). In contrast, deletion of either *rsbU* or *rmlA* produced differences in the levels of crystal violet staining measured over time. For the *rsbU* deletion strain, the biomass was higher than that of WT_1031_ at 36 and 48 h (Fig. 4A). For the *rmlA* mutant strain, biofilm formation was reduced at 36 and 48 h (Fig. 4A). These findings were in agreement with the average number of cells adherent per FOV that were visualized (Fig. 4B to G) and quantified following scanning electron microscopy (Fig. S4A and B). Therefore, we concluded that two genes that impact biofilm formation are mutated in WT_1030_: *rsbU* and *rmlA*. By constructing a double *rsbU rmlA* deletion strain in the WT_1031_ background, we established that the impact of the *rmlA* mutation dominated the moderate increase in biofilm observed when *rsbU* was deleted alone (Fig. 4A, E, and G).

### When sigma B is inactive, cell adherence increases.

Deletion of *rsbU* enhances biofilm formation, whereas deletion of *rmlA* decreases biofilm formation. RsbU is an upstream positive regulator of SigB (23); therefore, one possible interpretation of our data is that deletion of *rsbU* decreases transcription of the SigB regulon, leading to an increase in *rmlA* transcription. While an effect of SigB on transcription of *rmlA* has not been reported, this hypothesis would explain the enhanced biofilm capability of the *rsbU* mutant and decreased biofilm levels in the double *rsbU rmlA* strain and in the *rmlA* single mutant. Therefore, we first tested if the impact of mutating *rsbU* on biofilm formation manifests as a consequence of SigB inactivation. If our hypothesis was correct, then deletion of *sigB* should phenocopy the *rsbU* mutation.

**FIG 5:**
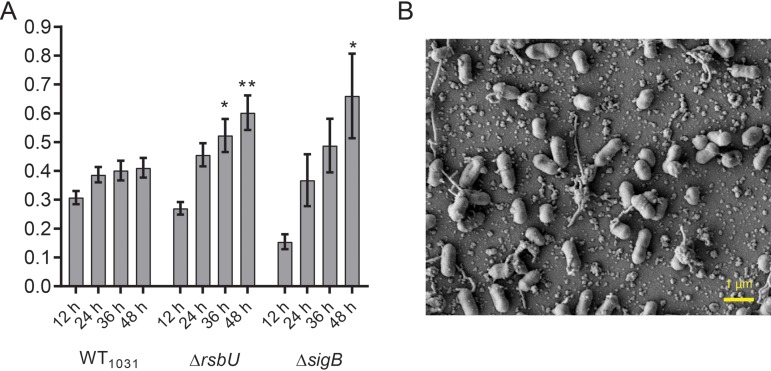
SigB influences biofilm formation by L. monocytogenes EGDe. (A) The biomasses of WT_1031_, WT_1031_ Δ*rsbU* (LSW1028), and WT_1031_ Δ*sigB* (LSW1026) strains that were adherent to the substratum were quantified. The samples were incubated at 30°C for the time points indicated. The values presented for WT_1031_ and WT_1031_ Δ*rsbU* are reproduced from Fig. 3 and 4. The means from ≥4 experiments are presented for the WT_1031_ Δ*sigB* strain. The error bars are the standard errors of the means. The data were analyzed by one-way ANOVA comparing with WT1031. *, *P* ≤ 0.05; **, *P* ≤ 0.01. (B) The biomass adherent to the substratum was imaged using scanning electron microscopy for WT_1031_ Δ*sigB*. The representative image shown was taken at the midpoint of the peg after 48 h of incubation.

We constructed a *sigB* deletion in WT_1031_, examined the level of chitinase activity, and assessed the impact on biofilm formation. As expected, the *sigB* deletion strain did not display chitinolytic activity (Fig. 2) (16). During biofilm formation, the *sigB* deletion strain was initially observed to have a lower level of biomass adherent to the pegs than the parental WT_1031_ strain. However, the value surpassed that of the parental strain at later time points (Fig. 5A). As suggested by the crystal violet staining in Fig. 5A, the *sigB* and *rsbU* strains were shown to have similar numbers of cells attached per FOV when the samples were imaged by scanning electron microscopy (SEM) (Fig. 5B; Fig. S4A and B). Together, these findings are consistent with the conclusion that the impact of the SNP in *rsbU* on biofilm formation was due to a reduction in *sigB* activity.

We next reasoned that if the reduction of SigB activity in the *rsbU* mutant impacted *rmlA* transcription, this would manifest as an alteration in l-rhamnose decoration of the wall teichoic acid (WTA). This is because RmlA is an enzyme in the TDP-l-rhamnose pathway. TDP-l-rhamnose is used for the synthesis of cell wall carbohydrates (24–26) and for the decoration of WTA in L. monocytogenes (27, 28). Therefore, we extracted WTA from the *sigB* and *rsbU* mutants and compared the apparent molecular mass with the WTA extracted from the *rmlA* mutant. These analyses showed there was no gross difference in the apparent molecular mass of WTA produced by the *sigB* and *rsbU* strains compared with that of the parental strain, at either a lower or higher position, as would be expected for material with fewer or more rhamnose moieties, respectively. In contrast, for the *rmlA* mutant, the molecular mass of WTA extracted was lower than that observed for WT_1031_. The mobility of the WTA extracted from the *rmlA* mutant was comparable to that of the WTA extracted from EGDe isolate WT_1030_ (Fig. S5). Therefore, taking these data together, it is unlikely that *rsbU* or s*igB* is mediating the impact on biofilm formation via *rmlA* and its impact on WTA decoration.

### Sugar decoration of wall teichoic acids alters adhesion properties of L. monocytogenes.

**FIG 6:**
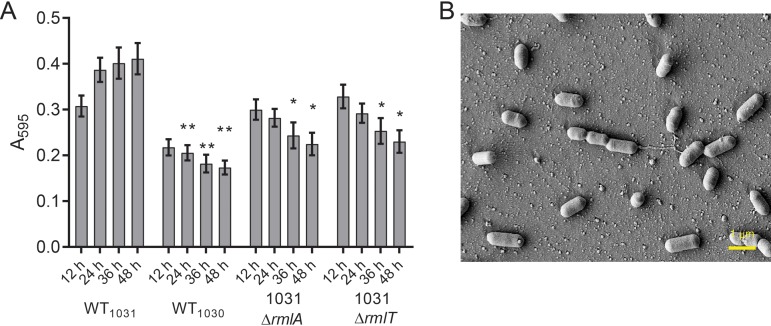
Decoration of the wall teichoic acid with L-rhamnose is needed for cell adhesion by L. monocytogenes EGDe. (A) The biomasses of WT_1031_, WT_1030_, WT_1031_ Δ*rmlA* (LSW1040), and WT_1031_ Δ*rmlT* (LSW1039) strains that were adherent to the substratum were quantified. The samples were incubated at 30°C for the time points indicated. The values presented for WT_1031_, WT_1030_, and WT_1031_ Δ*rmlA* are reproduced from Fig. 3 and 4. The means from ≥4 experiments are presented for the WT_1031_ Δ*rmlT* strain. The error bars are the standard errors of the means. The data were analyzed by one-way ANOVA comparing with WT1031. *, *P* ≤ 0.05; **, *P* ≤ 0.01. (B) The biomass adherent to the substratum was imaged using scanning electron microscopy for WT_1031_ Δ*rmlT* after 48 h of incubation. The representative image shown was taken at the midpoint of the peg.

RmlA is the first enzyme in the pathway that catalyzes the conversion of d-glucose-1-phosphate into TDP-l-rhamnose (27). We wanted to confirm if deletion of *rmlA* had an impact on biofilm formation due to the lack of the l-rhamnose moiety on WTA or if TDP-l-rhamnose was used in the synthesis of a different polymer. To do this, we constructed a derivative of WT_1031_ that still produced TDP-l-rhamnose but lacked the glycosyltransferase, RmlT, which is responsible for the transfer of TDP-l-rhamnose onto ribitol phosphate (27). Biofilm formation was measured for the 1031 Δ*rmlT* strain every 12 h and found to be more comparable to that of the 1031 Δ*rmlA* strain than to that of the WT_1031_ strain (Fig. 6A). Using SEM imaging to visualize the attached biomass, the 1031 Δ*rmlA* and 1031 Δ*rmlT* strains were shown to have similar numbers of cells attached per FOV (Fig. 6B; Fig. S4A and B). Therefore, as presence of the TDP-l-rhamnose pool in the *rmlT* mutant strain was not sufficient to allow biofilm formation, these findings suggest that decoration of WTA with l-rhamnose is needed for cell adhesion to the substratum. It is also possible that decoration of WTA with l-rhamnose is needed to promote the formation of clusters of the bacteria, but further analysis would be needed to determine this conclusively.

## DISCUSSION

To study biofilm formation by the Gram-positive pathogen Listeria monocytogenes, we chose an approach that was based on the hypothesis that diverged stocks of the EGDe wild type may contain mutations that could impact biofilm formation. We proposed that identifying the mutations would allow us to link genotype with phenotype and thereby gain insights into the mechanisms underpinning biofilm formation in this pathogen. We sourced four EGDe isolates, checked planktonic growth, and identified differences in chitinase activity. Using next-generation sequencing technologies, we sequenced the genomes of the four isolates and identified genomic variations. Some features of the genomic sequencing data were able to be readily connected to phenotypic differences displayed by the four EGDe isolates. For example, RsbU is an upstream regulator of SigB activity (17, 29, 30), and in WT_1030_ and WT_1033_, a frameshift mutation that leads to the premature termination of translation is contained within *rsbU* (*lmo0892*) (Table 2). A consequence of the *rsbU* mutation may be that SigB is not activated, and transcription of the genes in its regulon will not be triggered (30); although, there is evidence showing that SigB retains partial activity in an RsbV mutant background (31). The presence of the SNP in *rsbU* correlated with the reduction of chitinolytic activity observed for WT_1030_ and WT_1033_ (Fig. 2). Additionally, WT_1030_ contains a nonsense SNP within the *rmlA* (*lmo1081*) coding region. RmlA is the first enzyme in a four-step reaction resulting in the synthesis of TDP-l-rhamnose (27), which is a substrate to transfer l-rhamnose onto the ribitol phosphate backbone of wall teichoic acid. The nonsense SNP in *rmlA* is predicted to disrupt TDP-l-rhamnose production, resulting in a strain that carries WTA without the l-rhamnose decoration. The presence of this mutation correlates with the lower molecular weight of the WTA extracted from WT_1030_.

We adapted and implemented a robust method of assessing biofilm formation by the four EGDe isolates. The biofilm formed under these conditions did not typically appear to generate an obvious extracellular matrix; when viewed by microscopy, the biomass appeared to be isolated cells or small clusters that were adherent to the surface. This is different from the honeycomb arrangement of L. monocytogenes cells seen in some biofilms (32) but comparable to that in other studies where cells have been observed as an attached monolayer (33). Through our analysis, we identified one strain (named WT_1030_) that displayed a defect in biofilm formation. Having ruled out that differences in growth or motility caused the differences in the biofilm formation we observed, we used the details from the next-generation sequencing analysis to link *rmlA* to surface adhesion and biofilm formation. As detailed above, RmlA is needed for TDP-l-rhamnose production, and through assessing biofilm formation lacking RmlT, we were able to determine that the lack of l-rhamnose decoration of wall teichoic acid was the factor influencing biofilm formation rather than the loss of TDP-l-rhamnose production *per se*. The defect in biofilm formation appeared to be due to reduced cell surface adhesion. Our findings are consistent with data derived from a global transposon screen of L. monocytogenes isolate 568 which identified *lmo1080* (*rmlT*) as needed for biofilm formation at low temperature (34). In addition, they are in line with experiments that uncovered wall teichoic acids as a major polysaccharide present in the L. monocytogenes biofilm matrix (35). However, exactly how the l-rhamnose decorated wall teichoic acid aids cell surface interaction remains unknown.

We also strengthened the already identified connection between *sigB* and biofilm formation and in so doing, reinforced the need to obtain dynamic data when analyzing biofilm formation using a microtiter plate-based assay (7, 36). SigB was previously found to promote biofilm formation (37, 38). However, here, for the *sigB* deletion strain, a defect in biofilm formation at early time points culminated in an enhanced level of biofilm produced at later time points. We therefore conclude that SigB appears to suppress transcription of genes involved in biofilm formation, perhaps those directly linked with matrix synthesis, as deletion resulted in greater adhesion and more extracellular material being deposited and visible by SEM analysis.

### Concluding comments.

The use of laboratory reference strains was initially focused on allowing the cooperation of research groups around the world (8). It provides a baseline of commonality to compare observations and accelerate the progression of research. Although this goal has been accomplished, the approach also allows seemingly identical isolates of bacteria to independently evolve in different laboratories (9, 11). Using a comparative sequencing approach, we have uncovered variations in the genomes of EGDe isolates used in laboratories across the world. Moreover, we have reinforced the importance and necessity of obtaining whole-genome sequencing data to ensure that strains do not contain inadvertent mutations when a new isolate is used in research settings.

## MATERIALS AND METHODS

### Growth media and additives.

Brain heart infusion (BHI) medium (237500; BD Biosciences) was used for propagating L. monocytogenes strains. Strains were routinely grown either in liquid BHI medium, on BHI medium solidified with 1.5% (wt/vol) select agar, or in liquid modified Welshimer’s broth (MWB) (6.56 g/liter KH_2_PO_4_, 16.39 g/liter Na_2_HPO_4_, 0.41 g/liter MgSO_4_·7H_2_O, 10 g/liter glucose, 0.088 g/liter ferric citrate, 0.1 g/liter leucine, 0.1 g/liter isoleucine, 0.1 g/liter valine, 0.1 g/liter methionine, 0.1 g/liter arginine, 0.1 g/liter cysteine, 0.6 g/liter glutamine, 0.5 mg/liter riboflavin, 1.0 mg/liter thiamine, 0.5 mg/liter biotin, and 0.005 mg/liter lipoic acid). Starter cultures were prepared by inoculating a single colony of L. monocytogenes grown on BHI agar into 5 ml of BHI medium, which was grown with shaking. The growth medium was supplemented with selective antibiotics (100 μg/ml ampicillin [Amp], 5 μg/ml erythromycin [Ery], or 50 μg/ml X-Gal [5-bromo-4-chloro-3-indolyl-d-galactopyranoside]) during cloning and the construction of mutant strains as required.

### Strains, plasmids, and primers.

Complete details of the strains, plasmids and primers used in this study are provided in Tables S1 to S3 in the supplemental material.

### Growth measurement.

To follow the growth of L. monocytogenes strains, starter cultures were grown at 37°C for ∼20 h and inoculated in 100 ml of BHI medium at a starting optical density at 600 nm (OD_600_) of 0.05. The cultures were incubated in a water bath with shaking at 200 rpm, and the OD_600_ was measured every hour. Alternatively, growth over time was monitored using a plate reader (Synergy 2; BioTek Instruments). The starting cultures were subcultured in MWB at an initial OD_600_ of 0.01 in 200 μl per well in a round-bottom polystyrene 96-well plate. The OD_600_ was measured every hour during incubation at 30°C for 48 h without shaking.

### Motility.

Semisolid (0.3% [wt/vol]) agar was prepared in BHI medium or MWB. Starter cultures for each strain were grown at 30°C for up to 48 h. To seed the strains, the OD_600_ of starting cultures was normalized to 1.0, and 1 μl of the normalized culture was stabbed into the center of a semisolid agar plate. A negative control, the nonmotile strain EGDe Δ*flaA* (13), was included. The seeded semisolid agar plates were incubated at 30°C, and after 24 and 48 h of incubation, images were captured using a digital single-lens reflex (DSLR) camera (Nikon D3200 with Nikkor 18- to 55-mm lens). Quantification of motility was performed by measuring the diameter of the zone occupied by the cells. For each sample, the diameter of the swarm was measured at two positions. The average of the two values was used for further statistical analysis.

### Chitinase activity.

Chitinase activity was tested as described previously (18). Starter cultures were grown at 37°C for ∼20 h. The cultures of the strains were normalized to an OD_600_ of 1.0, and 10 μl was spotted onto an LB agar plate supplemented with colloidal chitin at a final concentration of 2% (wt/vol). The plates were then incubated at 30°C for 24 and 48 h prior to imaging using a DSLR camera (Nikon D3200 with Nikkor 18- to 55-mm lens).

### Cell wall teichoic acid analysis.

Extraction of the cell wall teichoic acids from L. monocytogenes was performed as described previously (39). Starter cultures were grown at 37°C for ∼8 h and inoculated in 50 ml of MWB at an initial OD_600_ of 0.01, which was incubated at 30°C for ∼17 h with shaking at 200 rpm. The cells were harvested by centrifugation at 3,800 × *g* for 10 min. The cell pellet was washed with 20 ml of MES buffer [50 mM 2-(*N*-morpholino)ethanesulfonic acid, pH 6.5] and centrifuged at 3,800 × *g* for 10 min. The cell pellet was resuspended in 1 ml of MES buffer supplemented with 4% (wt/vol) SDS and boiled at 99°C for 1 h. The SDS-treated cells were harvested by centrifugation at 17,000 × *g* for 10 min. The cell pellets were washed twice with MES buffer containing 2% (wt/vol) NaCl, rinsed with MES buffer, and resuspended in 1 ml of MES buffer with 0.4 g acid-washed glass beads (≤106 μm, catalog number G4649-500G; Sigma-Aldrich) per sample. The cells were lysed by vortexing at the highest speed for 10 min with the tube lying horizontally. The glass beads were discarded after centrifugation at 1,000 × *g* for 5 min, and the cell lysate was harvested for the following steps. The proteins in the samples were digested with 20 μg/ml proteinase K (03508811103; Roche) in 20 mM Tris-HCl (pH 8.0) at 50°C for 2 h. After centrifuging at 17,000 × *g* for 10 min, the pellet was treated with 1 ml of 0.1 M NaOH for 17 h with shaking at 1,200 rpm at 25°C on Thermomixer R (Eppendorf). The supernatant was harvested by centrifugation at 14,000 × *g* for 15 min, and 0.1 ml of 1 M HCl was added to each sample. The liquid was dialyzed into Milli-Q water using a 1-kDa dialysis membrane (132105; Spectrum). The dialyzed samples were dried with a SpeedVac (RVC2-25 with CT02-05; Christ). Each sample was resuspended with 100 μl of WTA loading buffer (20 mM Tris-HCl, 20 mM Tricine, 10% [vol/vol] glycerol) for further analysis by native polyacrylamide gel electrophoresis. The gel was rinsed with Milli-Q water and stained with alcian blue staining solution (5% [vol/vol] acetic acid, 30% [vol/vol] ethanol, and 1 mg/ml alcian blue 8GX) for 1 h. An image of the stained gel was taken after incubation in destaining solution (5% [vol/vol] acetic acid, 30% [vol/vol] ethanol) for 20 min.

### Biofilm formation.

Starter cultures were grown at 37°C for ∼20 h, and the OD_600_ was normalized to 0.01 in MWB. One hundred fifty microliters of the diluted cultures was subcultured in the Calgary biofilm device (catalog number 445497 for the lid, and catalog number 262162 for the plate; Nunc, Thermo Scientific) and incubated at 30°C for 12 to 48 h. The biomass of the biofilm formed was determined by crystal violet staining. The cultures were discarded by aspiration, each well was rinsed three times with 1.2 volumes of 1× phosphate-buffered saline (PBS; 8 g/liter NaCl, 0.2 g/liter KCl, 2.56 g/liter Na_2_HPO_4_·7H_2_O, 0.2 g/liter KH_2_PO_4_, pH 7.4), and cells were incubated with 1.3 volumes of 0.1% (wt/vol) crystal violet (diluted from 2.3% solution in Milli-Q water, HT901-8FOZ; Sigma-Aldrich) for 1 h at room temperature. The staining solution was aspirated, and the peg was washed with 1.5 volumes of 1× PBS three times. The biofilm was destained by incubating with 30% (vol/vol) acetic acid for 30 min at room temperature. The absorbance of the stained 30% (vol/vol) acetic acid was measured at a wavelength of 595 nm. For each replicate, the *A*_595_ of a medium-only control was used as the background reading.

### Scanning electron microscopy.

Biofilms formed on the pegs of the Calgary biofilm device were fixed for scanning electron microscopy (SEM) largely as described previously (40). The protocol involved two different stages of fixation, critical-point drying and sputter coating with platinum prior to final imaging. The biofilm-coated pegs were first rinsed with 1× PBS three times and fixed with 200 μl per well of primary fixative for 2 h at room temperature. The primary fixative comprised 2.5% (vol/vol) glutaraldehyde, 4% (wt/vol) paraformaldehyde, 75 mM l-lysine, and 0.075% (wt/vol) alcian blue in 1× PBS. Next, the pegs were removed from the Calgary biofilm device by using diagonal pliers. A secondary fixation step was included after a brief wash with 1× PBS. The secondary fixative was composed of 1% (wt/vol) osmium tetroxide (diluted from 4% stock, 75632; Sigma-Aldrich). After 1 h of secondary fixation, the biofilms were treated with a gradient ethanol series (50%, 70%, 90%, and 99.9% [vol/vol]). The biofilm-coated pegs were transferred into a chamber to be critical-point dried. Biofilm-coated pegs were stuck onto a 25-mm sample stub (AGG3023; Agar Scientific) with carbon stickers (AGG3303; Agar Scientific) and conductive carbon double-sided tape (AGG3939; Agar Scientific). The sample stub carrying the biofilm-coated pegs was sputter coated with 25-nm-thick platinum to create a conductive surface. The biofilms were imaged with field emission SEM (JSM-7400f; Jeol). All images were taken with 5 kV detected by a lower secondary electron (LEI) detector. The cells in each image were counted manually with a cell counter plug-in in ImageJ.

### Electrocompetent cells.

To insert plasmids into L. monocytogenes strains, electrocompetent cells were prepared as described previously (41). Plasmid DNA (1 μg) was gently mixed with 50 μl of electrocompetent cells before incubating them on ice for 10 min. The cells were transferred into a chilled electroporation cuvette (1652089; Bio-Rad) and electroporated at 10 kV/cm, 400 Ω, and 25 μF. A recovery medium, 1 ml of 0.5 M sucrose-supplemented BHI medium, was gently added to each electroporation reaction. Following incubation at 30°C for 90 min without shaking, 150 μl of the cell suspension was plated on a BHI agar plate supplemented with antibiotics as required.

### Construction of deletion strains.

In-frame deletions of protein-coding regions on the chromosome were introduced by the pMAD-based approach (42). First, the pMAD-based plasmid was modified such that it could be used for allelic exchange. Both upstream and downstream regions of the gene to be deleted were either amplified and fused with a KpnI restriction enzyme site using PCR or were synthesized commercially. The modified DNA sequences were first inserted into an intermediate cloning vector, pUC19 or pUC57, prior to ligation into pMAD. The pMAD vector containing the required insert was introduced into the desired parental strain. The recovered cells were spread on BHI agar plates supplied with 5 μg/ml Ery and 50 μg/ml X-Gal and incubated at 30°C for 72 h. The resultant colonies were collected, inoculated in BHI medium containing 5 μg/ml Ery, and incubated at 39°C with shaking at 200 rpm for 17 h. The cultures were serially diluted to a factor of 10^−6^ and isolated on 5 μg/ml Ery- and 50 μg/ml X-Gal-supplemented BHI agar plates that were incubated at 39°C for 48 h. Blue-colored colonies were used to inoculate liquid BHI medium, and the cells were incubated at 30°C for 17 h without shaking and then for 4 h with shaking at 200 rpm. The cultures were serially diluted to a factor of 10^−6^ and isolated on 50 μg/ml X-Gal-supplemented tryptic soy agar plates. The plates were incubated at 37°C for 72 h to allow the formation of white colonies. Each white colony was inoculated in 5 ml of BHI medium and incubated at 37°C with shaking at 200 rpm for ∼17 h. Deletions were confirmed using PCR and DNA sequencing.

### Statistical analysis.

GraphPad Prism 7 was used to generate plots and analyze data. Statistical analyses of the data were performed by one-way analyses of variance (ANOVAs) with Dunnett’s multiple-comparison tests.

### Genome sequencing.

Genomic DNA was extracted from starter cultures incubated at 37°C for ∼17 to 20 h. The cells were harvested by centrifugation at 3,500 × *g* for 10 min, and the cell pellet suspended in 180 μl of enzymatic lysis buffer (20 mM Tris-HCl, 2 mM EDTA [pH 8.0], 1.2% [vol/vol] Triton X-100 containing 20 mg/ml lysozyme). The cells were lysed at 37°C for 30 min after which the cell lysate was applied to the DNeasy blood and tissue kit (69504; Qiagen). The final product was eluted in water and stored at −20°C.

Illumina next-generation sequencing was performed by the Genome Sequencing Unit at the Tayside Centre for Genome Analysis. The DNA was quantified using the QuBit 2.0 DNA kit, and 1 μg of DNA was sheared into 300-bp fragments using a Covaris M220 focused ultrasonicator. Paired-end libraries were generated using the Illumina TruSeq DNA sample preparation guide and sequenced using the Illumina MiSeq reagent kit v2 on the Illumina MiSeq platform.

### Sequence analysis.

The list of the single nucleotide polymorphisms (SNPs) was acquired by aligning the reads to the published genome (NC_003210). The sequence data were analyzed using MiSeq Reporter, alignment to the reference genome was conducted using Burrows-Wheeler Aligner (43), and variant calling to identify SNPs was performed using the Genome Analysis Toolkit UnifiedGenotyper (44). To determine if the A118 prophage was integrated, genome assemblies of strains WT_1030_ and WT_1032_ were carried out using the BugBuilder (45) pipeline, using SPAdes (46) for contig assembly (version 3.13.1, coverage cutoff = 5, kmer size = auto, and “careful” mode enabled). Scaffolding was carried out with the Mauve Contig Mover (47) (version 2.4.0) with NC_003210.1 as a reference sequence, followed by automated gap closure using Pilon 1.23 (48). Annotation of the assembled sequences was carried out using Prokka 1.13.4 (49). Assembled genomes were aligned against NC_003210.1 using pairwise comparisons with NCBI BLAST (50) (blastn version 2.7.1, E value cutoff = 0.01), and alignments were visualized using the Artemis comparison tool (51).

Additional bioinformatics analysis performed in this study used CLC Main Workbench 8 to organize the DNA sequences. Basic Local Alignment Search Tool (BLAST) was used to align sequences of nucleic acids (https://blast.ncbi.nlm.nih.gov/Blast.cgi). The ExPASy translation tool was used to assess the impact of the mutations on the protein sequences (https://web.expasy.org/translate/). The alignment of the protein sequences was generated by Clustal Omega (52).

### Data availability.

Sequence data have been deposited in the European Nucleotide Archive under study accession numbers PRJEB35200 and ERZ1188925.

## Supplementary Material

Supplemental file 1
