# Supplementary material for: Genomic Differences between Listeria monocytogenes EGDe Isolates Reveal Crucial Roles for SigB and Wall Rhamnosylation in Biofilm Formation
Source: J Bacteriol. 2020 Mar 11;202(7):e00692-19. doi: 10.1128/JB.00692-19 (PMC7167478; doi:10.1128/JB.00692-19)
Supplement: Supplemental file 1 [file JB.00692-19-so001.pdf]

Supplemental Material

Genomic differences between *Listeria monocytogenes* EGDe isolates reveals crucial roles for SigB and wall rhamnosylation in biofilm formation.

Chih-Yu Hsu [1], Lynne Cairns [1#], Laura Hobley [1,2#], James Abbott [3], Conor O'Byrne [4], Nicola R. Stanley-Wall [1]

[1.] Division of Molecular Microbiology, School of Life Sciences, University of Dundee, Dundee, UK DD1 5EH

[2.] Current address: School of Biosciences, University of Nottingham, Sutton Bonington Campus, Loughborough, Leicestershire, LE12 5RD

[3.] Data Analysis Group, Division of Computational Biology, School of Life Sciences, University of Dundee, Dundee, UK DD1 5EH

[4.] Bacterial Stress Response Group, National University of Ireland, Galway, Ireland.

# These authors contributed equally and are listed alphabetically by surname

To whom correspondence should be addressed:

Prof. Nicola R. Stanley-Wall

Division of Molecular Microbiology, School of Life Sciences, University of Dundee, Dundee DD1 5EH.

Email: n.r.stanleywall@dundee.ac.uk

Keywords: *Listeria monocytogenes*, biofilm formation, Sigma B, rhamnose, cell wall teichoic acid

Materials and Methods

Synthetic DNA constructs

The regions of DNA used to construct in frame deletions in *rsbU*, *sigB* and *lmo0184* were generated using DNA synthesis techniques. The sequences synthesised are detailed below where the underlined sequence indicated restriction sites.

For deletion of *rsbU* (pNW1454):
GGATCCGAGTACTTTCGGCGATGGACCTTGAAAGTGGTCTGGAAAAACTTAAACAGGAATTGGGGGAATGAA
CATGACATTCCAATCCTGTGTAAAGATAATTAATGAATGGGATATTGTAGCTGCAAGGCAACTAGGTAGAAAA
ATATCCAAAGAAATTGGCTTTGGAACCGTTGACCAAGCAAGAATTACAACTGCCATCAGTGAATTAGCTAGAA
ATATTTTCCTTTATGCTGGACGAGGAGAAATCTGTATCGAAAAAGTAAGTGAATCTGGCAAACAAGGTATGAT
TATTGTTGCCAAAGACAAAGGTCCAGGTATTGTAGACATAAGAAAAGTAATGCAAGATGGTTATACAACATCA
GGTGGTCTTGGAGCAGGTCTTCCAGGAGTCAAACGTTTAATGGACAGTTTTGATATTGAATCCAGTATTGAAG
GCGATTCTAAAGGAACGGTAATTACAACAACGAAATGGGTTCGGTAAGGAGAGCTAGAAAAAAATGGAACG
AACTAAATAAGAAGAAGTGAAGACGTACCTCATTCTTTAGAGAGCATTTACTGGTGAGTAAACGTATTTAAAG
AGTGGAGCGTTTTTATTTTTGAATGTTTTAATTTTATTTGTTAGGGTAAAATCGACAGTATACTTAAAATTAGAT
GGGGTGAAGTGATGAATATTAGTATAGAAATAAAAGAACGTGATACTGACCACATAGACATATTTGTTGCTG
GGGAGATCGATGCTTATACAGCGCCAAAGGTAAAAGAAGCATTAGAAGTATATCAAGTTAAAGAGGGTATTG
TACTTCGGATCGATTTAACAGAAGTGAGTTACATGGATAGCACCGGATTAGGCGTATTTGTAGGAGCTTTCAA
AAGCTTACGTCAACGCCAAAGTGAACTTGTCTTGTTTGGTTTAAGCGACCGACTTTTCCGATTGTTTGAAATCA
CAGGATTGTCAGATATCATCGAAATCAAAAATGTAGAGGGTGAAATGAATGGCAACAATAGATCT

For deletion of *sigB* (pNW1455):
GGATCCAATGTAGAGGGTGAAATGAATGGCAACAATGCATGACAAAATTACATTACAACTTCCTGCCAAGCCT
GAATATGTAAGTTTAGGTAGACTTTCATTATCAGGAATTGCAAGTCGCGCAGGATTTTCTTATGAAGCAATTGA
AGATTTGAAAATAGCTGTAAGTGAAGCCATCACTAATTCCGTAAAGCATGCATTTAAAGGGGAAGAAGATGG
CGAAATCACAGTCGAATATCTTATTTATGAAGATAAGCTAGAAGTTCGTGTTTCCGATAACGGCACAAGCTTC
GACTTAGAAACCCGTAAACAAGAAATTGGCCCATATGATGTGGGCGAGGATGCGGAGATGATGCGTATCGGT
GGGCTAGGTTTATTTTTAATTGAAACATTAATGGATGACGTGAAACTTTATTATGATGAAGGGGTTTCTGTCGT
AATGACCAAATATATTAATGAAAAGCAGGTGGAGGAGAATGCCAAAAGTATCTCAACCTGATAAAGAGCAGA
ATGAGGAAGTGGAGTAAATGAACAAGGCAGTTGAATCAAATAATTTATTTGTATTTCAACGTTCTAAAGCATT
ACAACAATACTGCGGGGATGTTTATTATACCCATGAAGACGAAAATGGTTTTCTGTATGTTCTTTCTGATGGAC
TTGGAAGTGGGCTCGAAGCTAATAGAGCGGCCAAAGCGACTGTTGACGCCATAAAAGAAGATATTCATGCAG
ATATTACTGATATGCTTGAAAAAGCGAACCAGGCTGTTTCAGGGCTTCGTGGTGCTGCGATAGCTATTATTAA
AGGTGACTACTTAACGAAGACACTTTATTATACTGGTATGGGTAATATTCGTTTTTATATGATTGGGATTGAAG
ATAAGCTTATTTTTCCGCTTTCAGGCTCTGGATTTTTGTCCGGTCGAAAACAGAAATATCGGTTGCAATCGTTTA
AATATAAACCAGACAGTAAGTTTTTAATGCATTCAGATGGACTTGTTCTTTCTCGAGATCT

For deletion of *lmo0184* (pNW1451):
GGATCCTAATTCAAGTAAACGATCAATTTATGCTTGGTGAAAATATCCTCGTTGCGCCAATCGTTCGCGAAGGC
CAAGCGAAGCGCCTAGTTCGTTTACCAAAAGGGCTTTGGTTTAATTATTGGACAAAAGAGCAGTTCGTGGGCG
GCGACTATATTATCGCAGACGCGCCGATTGATACCATGCCAATTTACATTAAAGCAGGAACAATTCTGCCAGTT
GGTAGCAGCGTTCAAAATACAAAAGAGACGCAAGAATTGACATTAGAAGTTTACTTAGACAGTGAGACAGCA

ACTGGTTACGTGTATAATGATGATGGGAAAAGTTACCAATATGAAAGTGGCGCAGTATCCAAAACAACACTCA
CAGCTACTTTCAAAAATGGCGAAGTACTAATAAATGCTACCCATCAAGGTGAAGCAAACTTGCAGCAAAAAGT
AACTACTATACAAGTGTTTGGAGAAAAAATAGACAAAATTACAAGGGCGGGAATTTAATCGATGAAACAAAT
CAAGTAAATCTCAGGAAAGCCACCATTTGCTAGGTGGCTTTTTTTGTATGCATCAAAAATTATAGTATAATAGA
AGGAAATGATTTTATTAGGAGGAATTCTCTTGCTATTTGATACGCATGTACACCTTAATGACGAGGCTTTTGAT
GATGATATAGAAGAAGTAATAAAACGCGCGCAGGAAAACGATGTGACTCGTATGGCCGTGGTGGGCTTTAAT
AAAGAAACAATTGACCGGGCGCTTGAATTAGCGGAGAAATATGATTTTATCTCATTAATCGTCGGCTGGCATC
CCACAGACGCTATTACCTTTACAGACGAAGATTTAGAATGGCTTCGTGATTTAGCACTGACACACCCAAAAGT
AGTTGCTTTAGGCGAAATGGGCTTAGATTATCACTGGGATACTTCGCCAAAAGAAACGCAATTCGAGGTTTTT
AGAAAACAAATCCGTTTAGCGAAAGAAGTGAATTTACCGATAGTTATTCACAACCGAGATCT

Table S1: Strains used in this study

**Strains**	**Genotype**	**Source/ Other names /Reference**
WT$_{1030}$	EGDe (stocked as LWS1030)	ANG882
WT$_{1031}$	EGDe (stocked as LWS1031)	ANG873
WT$_{1032}$	EGDe (stocked as LWS1032)	University College Cork
WT^{1033}	EGDe (stocked as LWS1033)	ATCC
DH-L1042	EGDe Δ*flaA* (stocked as LSW1035)	DH-L1042 (Grundling *et al.*, 2004)
LSW1024	WT1031 Δ*lmo0184*	This work
LSW1026	WT1031 Δ*sigB*	This work
LSW1028	WT1031 Δ*rsbU*	This work
LSW1039	WT1031 Δ*rmlT*	This work
LSW1040	WT1031 Δ*rmlA*	This work
LSW1051	WT1031 Δ*rmlA* Δ*rsbU*	This work

Table S2: Plasmids used in this study

Plasmid	Description
pUC19	Intermediate cloning vector
pUC57	Intermediate cloning vector
pMAD	Vector for constructing in-frame deletion in *L. monocytogenes*
pNW1451	pUC57 Δ*lmo0184*
pNW1454	pUC57 Δ*rsbU*
pNW1455	pUC57 Δ*sigB*
pNW1457	pMAD Δ*rsbU*
pNW1458	pMAD Δ*sigB*
pNW1459	pMAD Δ*lmo0184*
pNW1901	pUC19 Δ*rmlA*
pNW1904	pUC19 Δ*rmlT*
pNW1905	pMAD Δ*rmlT*
pNW1907	pMAD Δ*rmlA*

Table S3: Primers used in this study

Primer	Sequence (5'-3') [a]	Use
NSW2027	AGTGGTTTGCCGCTCGTTCG	Screening for Δ*lmo0184*
NSW2028	AATTCCGCCTACTTCTTCGG	
NSW2030	GTAACTGGGATACAGCCAGC	Screening for Δ*rsbU*
NSW2031	ACATATTCAGGCTTGGCAGG	
NSW2032	TTGTTTGGTTTAAGCGACCG	Screening for Δ*sigB*
NSW2033	CACAAAGTGGTGATTCTAGG	
NSW2301	CTTGGTGAATTCGACTATGATAATATCAAAATTTAC	Construct for Δ*rmlT*
NSW2302	TTTGGTACCTCTCATTATATCCTCCTAAAATAGATT	
NSW2303	AAAGGTACCCTTTAAGAATGGAGAGAAAAGAATGAA	
NSW2304	CGATTAGTCGACAAATAAAGACCAGTTACCGCATAA	
NSW2305	GTCGGCTAAGGTTTCAGCAA	Screening for Δ*rmlT*
NSW2306	GCTCAAAGCGTGGAGTATCT	
NSW2307	CAAATCCTGCAGGCCAAAAGAATTCTTTGATTATAG	Construct for Δ*rmlA*
NSW2308	AATGGTACCTTTCATTCTTTTCTCTCCATTCTTAAA	
NSW2309	TTAGGTACCAAATAAGAAGAGTGAGGCGTACTAATG	
NSW2310	GATATCGTCGACATTGCTCATTGTGCCTGCATAAGT	
NSW2311	CAAGTCACCGAGCATTAACC	Screening for Δ*rmlA*
NSW2312	CGGATCATCGTAGGCAATTC	
NSW2325	GATTTTATGCTAACTCATTGGTATCGCTCACGAG	
NSW2328	GCGGGATTAATTTTTCTGGGAAATGATGTGGTCC	
NSW2322	CGTAGGATCGATCCGATCCT	Sequencing pMAD
NSW817	CGTCATCTACCTGCCTGGA	
NSW2323	CTAAAGTTAATGGCAAAGCTCCTGCAAAATTAACG	Screening for Δ*rmlT*
NSW2324	CTTTCAACAATTTCCATTAGTACGCCTCACTCTTC	

a. The underlined sequences are restriction sites used for cloning purposes.

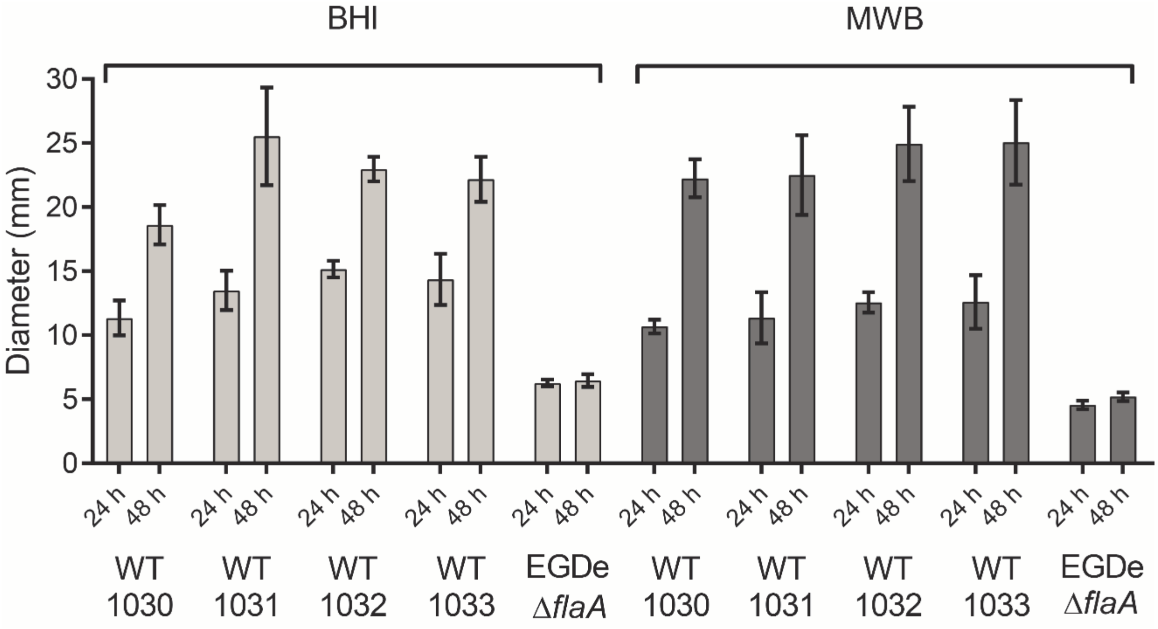

Figure S1: Quantification of motility of the four EGDe isolates. Quantification of motility of the four isolates assessed after 24 and 48 hours at 30ºC using BHI or MWB medium (representative images shown in Figure 1). The EGDe Δ*flaA* strain was used as a negative control.

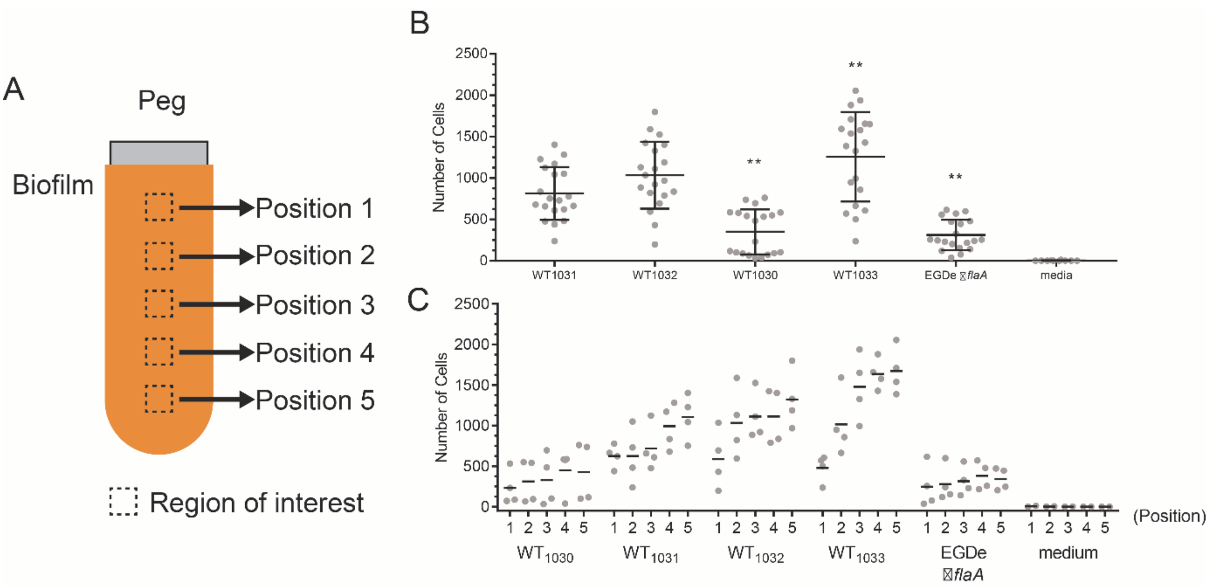

Figure S2: Quantification of cell adherence of the four EGDe isolates. The biomass adherent to the substratum was imaged using scanning electron microscopy. Representative image are shown in Figure 3. The number of cells adherent were quantified for all of the images taken. (**A**) Schematic of the imaging process showing the positions used to capture the images. (**B**) The mean number of cells per field of view adherent to the substratum for each of the strains is presented. The error bars represent the standard deviation. The data were analysed by one-way ANOVA comparing with WT1031 with "**" representing a p value of ≤0.01 (**C**) the same data presented in (**B**) are shown with respect to the image capture position. The bar represents the mean value for each position and strain.

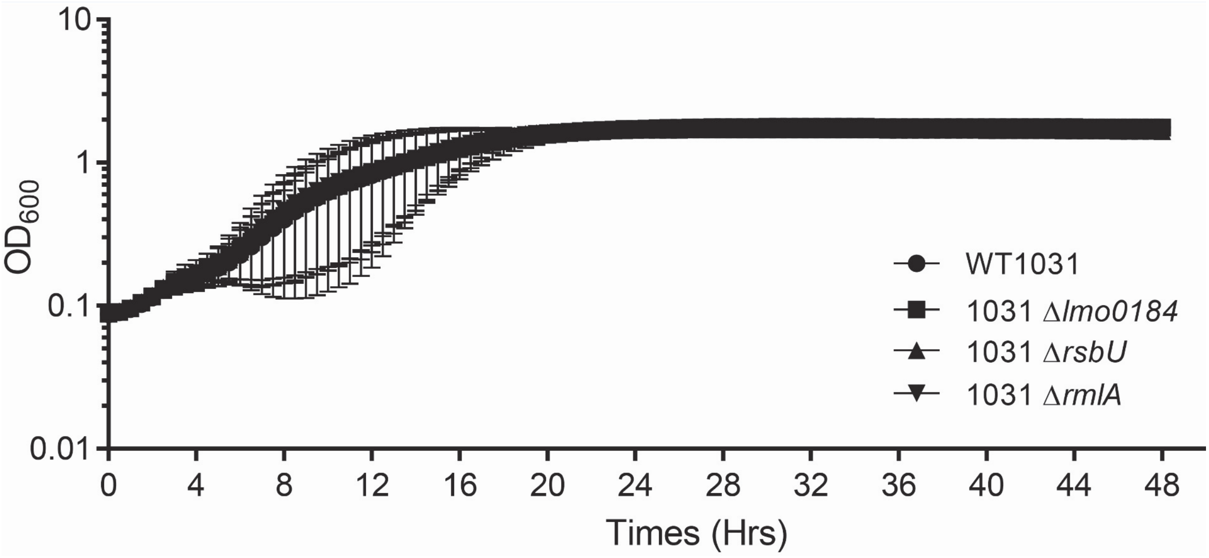

Figure S3: Analysis of growth of the single deletion strains. Growth in MWB medium for strains WT_{1031}, WT1031 Δ*lmo0184* (LSW1024), WT1031 Δ*rsbU* (LSW1028), WT1031 Δ*rmlA* (LSW1040) was assessed at 30ºC without shaking The value presented is the mean of 2 independent experiments and the error bars represent the standard deviation.

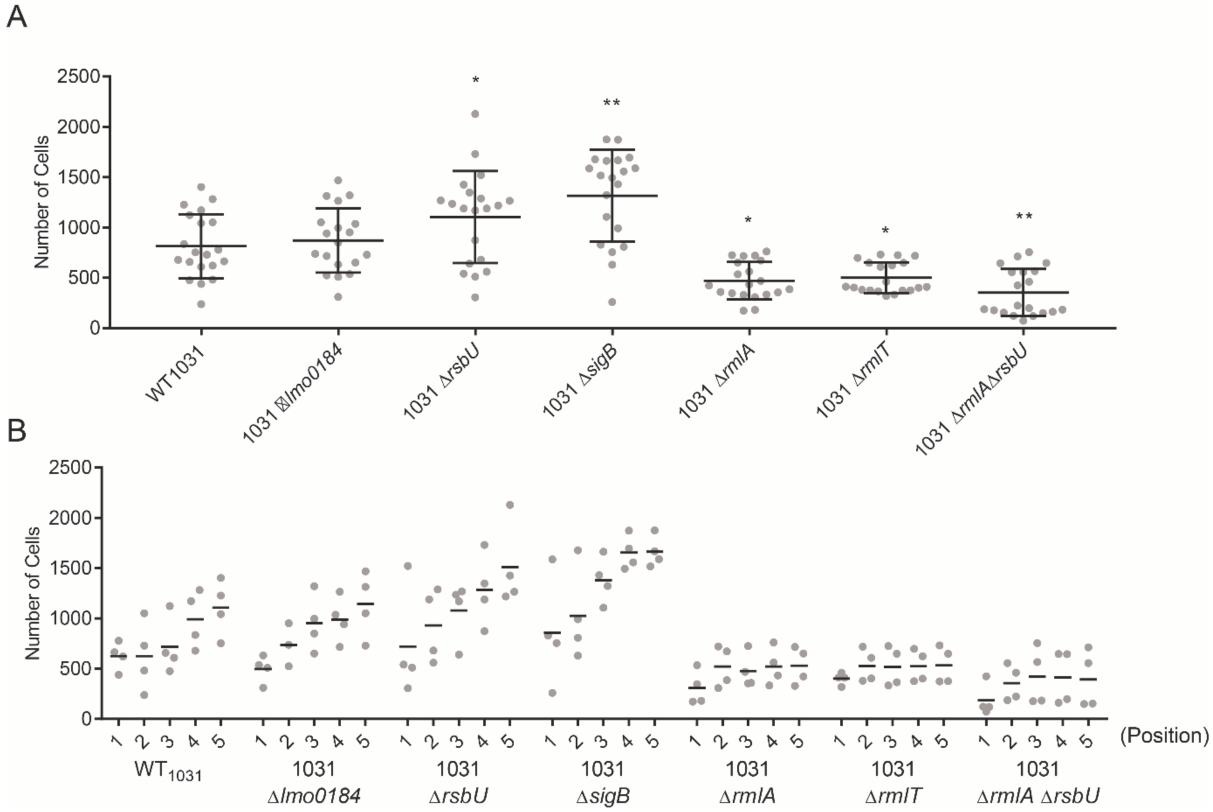

Figure S4: Quantification of cell adherence determined using scanning electron microscopy. The biomass adherent to the substratum was imaged using scanning electron microscopy. Representative images are shown in Figures 3, 4 and 5. The number of cells adherent were quantified for all of the images taken. A schematic of the imaging process showing the positions used to capture the images is shown in Figure S2A; (**A**) The mean number of cells adherent to the substratum for each of the strains is presented. The error bars represent the standard deviation. The data were analysed by one-way ANOVA comparing with WT1031 with "*" representing a p value of ≤ 0.05 and "**" representing a p value of ≤ 0.01; (**B**) the same data presented in (**A**) are shown with respect to the image capture position. The bar represents the mean value for each position and strain.

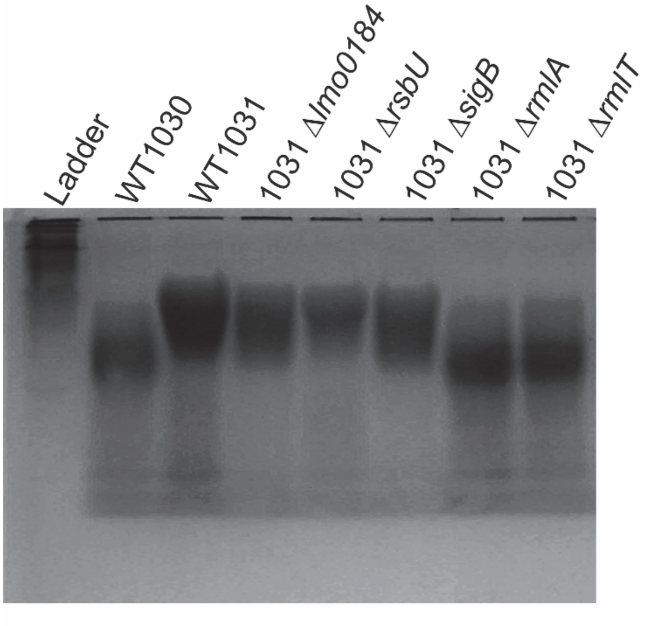

Figure S5: Cell wall extraction of *L. monocytogenes* EGDe mutant strains. Cell wall material was extracted from WT$_{1030}$, WT$_{1031}$, WT1031 Δ*lmo0184* (LSW1024), WT1031 Δ*rsbU* (LSW1028), WT1031 Δ*sigB* (LSW1026), WT1031 Δ*rmlA* (LSW1040) and WT1031 Δ*rmlT* (LSW1039), analysed by 20% (w/v) native-PAGE and visualised by staining with alcian blue.

Supplemental References

Grundling, A., Burrack, L. S., Bouwer, H. G. & Higgins, D. E. (2004). Listeria monocytogenes regulates flagellar motility gene expression through MogR, a transcriptional repressor required for virulence. *Proceedings of the National Academy of Sciences of the United States of America* **101**, 12318-12323.
